# Lyn‐mediated glycolysis enhancement of microglia contributes to neuropathic pain through facilitating IRF5 nuclear translocation in spinal dorsal horn

**DOI:** 10.1111/jcmm.17759

**Published:** 2023-05-02

**Authors:** Erliang Kong, Yongchang Li, Peng Ma, Yixuan Zhang, Ruifeng Ding, Tong Hua, Mei Yang, Hongbin Yuan

**Affiliations:** ^1^ Department of Anesthesiology, Changzheng Hospital Second Affiliated Hospital of Naval Medical University Shanghai China; ^2^ Department of Anesthesiology The 988th Hospital of Joint Logistic Support Force of Chinese People's Liberation Army Zhengzhou China

**Keywords:** glycolysis, interferon regulatory factor 5, Lyn, microglia, neuropathic pain, nuclear translocation

## Abstract

The pro‐inflammatory phenotype of microglia usually induces neuroinflammatory reactions in neuropathic pain. Glycometabolism shift to glycolysis can promote the pro‐inflammatory phenotype transition of microglia. The omics data analysis suggest a critical role for Lyn dysregulation in neuropathic pain. The present study aimed at exploring the mechanism of Lyn‐mediated glycolysis enhancement of microglia in neuropathic pain. Neuropathic pain model was established by chronic constriction injury (CCI), then pain thresholds and Lyn expression were measured. Lyn inhibitor Bafetinib and siRNA‐lyn knockdown were administrated intrathecally to evaluate the effects of Lyn on pain thresholds, glycolysis and interferon regulatory factor 5 (IRF5) nuclear translocation of microglia in vivo and in vitro. ChIP was carried out to observe the binding of transcription factors SP1, PU.1 to glycolytic gene promoters by IRF5 knockdown. Finally, the relationship between glycolysis and pro‐inflammatory phenotype transition of microglia was evaluated. CCI led to the upregulation of Lyn expression and glycolysis enhancement in microglia of spinal dorsal horn. Bafetinib or siRNA‐lyn knockdown intrathecally alleviated pain hyperalgesia, suppressed glycolysis enhancement and inhibited nuclear translocation of IRF5 in CCI mice. Also, IRF5 promoted the binding of transcription factors SP1, PU.1 to glycolytic gene promoters, and then the enhanced glycolysis facilitated the proliferation and pro‐inflammatory phenotype transition of microglia and contributed to neuropathic pain. Lyn‐mediated glycolysis enhancement of microglia contributes to neuropathic pain through facilitating IRF5 nuclear translocation in spinal dorsal horn.

## 1 INTRODUCTION

Neuropathic pain is a chronic condition that arises from injury or disease associated with peripheral or central nervous system which consequently affects the somatosensory nerves.^1^ Neuropathic pain is estimated to affect 7%–10% of the general population and severely reduces the life quality of patients suffering from chronic diseases, including diabetes, cancer, nerve injury, herpes zoster or HIV‐related neuropathies. Patients with neuropathic pain usually experience allodynia or hyperalgesia, with less satisfaction to routine drugs due to insufficient analgesia or severe side effects.^2^ The exact mechanism underlying the initiation and progression of neuropathic pain remains unclear and needs to be further explored.

The pathological basis of neuropathic pain is allodynia and hyperalgesia caused by synaptic remodelling during pain signal transmission. Long‐term chronic nerve injury can induce the release of various inflammatory factors to activate intracellular signal transduction pathways, resulting in the imbalance of facilitation and inhibition in pain transduction.^3^ Various nerve injury models that induce persistent pathological pain are widely used in animal experiments, such as chronic constriction injury (CCI) and spinal nerve ligation.^4^ Recent studies focused on the key influences of abnormal glycometabolism in neurons and supporting cells induced by chronic nerve injury. As is generally understood, energy metabolism is the core process in providing energy and substrates for signal transmission and cell responses, and glycolysis is the basis activity of cell energy metabolism.^5^ Microglia are widely present in central nervous system and involved in various neurophysiological processes including neuronal development, synaptic remodelling and immune response. Microglia participate in the chronic process of pain through various pathways, including the regulation of glutamate concentration in synaptic clefts through glutamate transporters, the controlled release of neurotransmitters such as serine, ATP and glutamate to regulate synaptic excitability, the altered stability of the synaptic microenvironment by certain metabolites, and the altered intercellular connections among neurons.^6^ Under stress, microglia can rapidly respond to a wide range of stimuli that threat the physiological homeostasis.^7^ Peripheral nerve injury in mice leads to a dramatic activation of microglia in the spinal dorsal horn.^8^ At the peak of inflammation induced by peripheral nerve injury, microglia preferentially use glycolysis as energy source, while they mainly rely on oxidative phosphorylation to obtain energy in the recession stage of inflammation to induce the conversion to anti‐inflammatory phenotype, accompanied with the changes in pentose phosphate pathway, amino hexanoic acid and glutamine hydrolysis pathway.^9^ Meanwhile, the intermediates produced in glycolysis also provide substrates for other biosynthetic pathway in cell growth and differentiation, some intermediates determine the activation or inhibition of intracellular signalling pathway, expression of key genes, transcription regulation and protein modification, even affect the fates of neurons or glial cells to some extent.^10^ Thus, the shift of glycometabolism to glycolysis may promote the pro‐inflammatory phenotype transition of microglia and induce the subsequent neuroinflammatory reactions.

Through analysing the database, we found that Lyn was closely related to glycometabolism and neuroinflammation in CCI model. Lyn, a key member of the non‐receptor Src family kinases (SFKs), plays a crucial role in immune inflammatory responses, cell proliferation, differentiation, apoptosis, migration and metabolism. The phosphorylated Lyn, which is the functional and activated type, can phosphorylate and modify the tyrosine residues of proteins to regulate their activities. Lyn is often regarded as a sensor of stress response for its strong influences on immune and inflammatory signalling pathways through downstream pathways.^11^ Studies have identified Lyn as an interferon regulatory factor 5 (IRF5) binding protein to regulate the TLR‐MyD88‐IRF5 pathway in mice suffering from autoimmune disease.^12^ IRF5 is highly expressed in macrophage subsets in peripheral system or microglia in central system, and plays a crucial role in the transcription process of pro‐inflammatory genes by binding to the interferon‐mediated response element domain of gene promoters or forming complexes with other transcription factors to promote transcription, especially the P65 subunit of NF‐κB.^13^, ^14^ Transcriptional activation of IRF5 is regulated by post‐translational modifications and nuclear translocation strictly to ensure appropriate expression of other key genes.^15^ SP1 and PU.1, the downstream transcription factors of IRF5, have been confirmed to bind to some glycometabolic gene promoter to promote the transcription of key glycometabolic genes, and this process may depend on IRF5 phosphorylation and nuclear translocation.^16^ Therefore, Lyn‐mediated nuclear translocation of transcription factors may be a key mechanism in inducing glucose metabolic reprogramming. The present study focused on observing the relationship between Lyn‐mediated glycolysis enhancement of microglia and neuropathic pain, and further exploring the exact mechanism of Lyn‐induced IRF5 nuclear translocation in glycolysis in vivo and in vitro, aiming at exploring the potential protective targets of neuropathic pain.

## 2 MATERIALS AND METHODS

### 2.1 Animals and drugs

Wild‐type C57BL/6J male mice (20–25 g) were provided by the Experimental Animal Center of Naval Medical University (Shanghai, China). All mice were housed in a pathogen free environment (24°C room temperature and 50% humidity) under a 12/12 h light/dark cycle, with food and water ad libitum. Maximal efforts were made to minimize the number of animals used in the study and reduce animal suffering. The study also acquired the permission from the animal ethics committee of Naval Medical University. Pentobarbital sodium was dissolved in normal saline and administrated intraperitoneally at 50 mg/kg for anaesthesia. Lyn inhibitor Bafetinib (Baf) (Selleck, INNO‐406) was dissolved in dimethyl sulfoxide (DMSO) and administrated intrathecally for once or continuous 5 days after CCI at 5, 25 and 50 μg in 10 μL. Glycolysis inhibitor 2‐deoxyglucose (2‐DG) (Sigma, No. D8375) was dissolved in normal saline and administrated intrathecally at 10 μg in 10 μL. For drugs intervention in cultured primary microglia, the final concentrations of Baf and 2‐DG were 5 and 10 mM, respectively. Lipopolysaccharide (LPS, Sigma, No. L2630) and interferon‐γ (IFN‐γ, Sigma, No. I4777) were used to activate the cultured primary microglia and the final concentrations were 1 and 10 ng/mL, respectively. siRNA targeting knockdown Lyn and IRF5 were purchased from OBiO Technology (Shanghai) company and the knockdown efficiency was successfully confirmed in mice and cultured primary microglia. siRNA‐lyn was administrated intrathecally for once or continuous 3 days after CCI. The scramble RNA (scRNA) was used as negative control.

### 2.2 CCI model establishment for neuropathic pain

CCI model for neuropathic pain was prepared by ligating the left sciatic nerve. After anaesthesia, an incision was made on the lateral surface of the right thigh, then the muscle layers were separated carefully to expose the sciatic nerve at the mid‐thigh level. Proximal to the sciatic nerve trifurcation, the nerve was freed of adhering muscle and three loose ligatures of 6‐0 chromic gut (W812, Ethicon Inc.) were tied 1 mm apart. The knots were slowly tightened until a brief twitch in the hind limb was observed. After nerve ligation, the muscle and skin were closed with 6‐0 silk. The same procedures were performed in sham mice except ligating the sciatic nerve.

### 2.3 Data acquisition and analysis

The transcriptome expression data were acquired from GSE186237 in the Gene Expression Omnibus (GEO) database. Hierarchical cluster analysis (HCA) and principal component analysis (PCA) were first conducted to explore the separation between CCI and non‐neuropathic‐pain group using the expression data. Differential expression genes (DEGs) were analysed between the groups using the DESeq2 package in R, with *p* < 0.05 and fold change (FC) ≥ 1.30. Gene Ontology (GO) annotation and Kyoto Encyclopedia of Genes and Genomes (KEGG) pathway enrichment analysis were performed with the R package clusterProfiler. In addition, the Spearman's rank correlation coefficient was calculated to construct gene co‐expression networks by Cytoscape, and *p* < 0.05 was considered to indicate statistical significance.

### 2.4 Measurements of mechanical and thermal pain thresholds

Mechanical and thermal pain thresholds were measured in a quiet room between 09:00 and 11:00. Mice were allowed to acclimatize to surroundings for 2 h in a set of Plexiglas™ cages with wire mesh floor. Von Frey filaments (North Coast Medical) of 0.07 and 0.4 g were applied to the middle surface of hindpaw with sciatic nerve distribution. The 0.07 and 0.4 g von Frey were used as low force values and high force values to evaluate pain allodynia and hyperalgesia, respectively. Mice were tested 10 times with an interval of 10 min. A positive withdrawal was scored if mice showed a brisk withdrawal. The number of positive withdrawal was counted and the percentage was calculated as the mechanical withdrawal frequency. Then mice were moved to the test cages with transparent glass plate for thermal pain thresholds measurement. After acclimatization, the heating device was placed under the hindpaw at certain heat intensity and a cut‐off time of 30 s to avoid tissue damage. A positive withdrawal was scored if mice showed a brisk withdrawal and the duration was regarded as the withdrawal latency. The withdrawal latency of the hindpaw was detected thrice for each mice at an interval of 5 min, and the mean value was taken as the thermal threshold.

### 2.5 Primary microglia culture

Newborn mice were sacrificed after anaesthesia and their spinal dorsal horn of lumbar segments were separated in a germ‐free environment. Subsequently the spinal dorsal horn were washed with cold PBS, cut into pieces, and digested with 0.25% trypsin at 37°C for 10 min. Then 10% FBS/DMEM was added to stop digestion and DNase I was added to the mixture at a final concentration 100 ng/mL to incubate at 37°C for 1 min. Mild pipetting was carried out for 10 times to break the mixture into single cell suspension. The suspension was centrifuged at 4°C for 5 min and the cell sediment was carefully collected. Then the cell sediment was re‐suspended with 10% FBS/DMEM containing recombinant mouse nerve growth factor (5 ng/mL) and recombinant murine fibroblast growth factor‐basic (10 ng/mL). After being filtered with a 70 μm strainer, the suspension was transferred to a T‐25 flask coating with poly L‐ornithine and cultured in an incubator (37°C and 5% CO_2_). The medium was exchange with fresh medium every day for 8 days, then the suspension was purified by shaking the flask at a speed of 200 rpm for 5 min to separate microglia from astrocytes. The medium was transferred into a new tube for centrifuging at 4°C for 5 min. The cell sediment was carefully collected and re‐suspended with fresh medium, and the number of cells was counted with an automated cell counter for seeding the purified microglia to dishes for functional study.

### 2.6 Western blot

Spinal dorsal horn samples and cultured primary microglia were collected to test the protein expressions. For total protein extraction, samples were sonicated in ice‐cold RIPA lysis buffer. Nuclear and cytoplasmic protein extraction was performed with NE‐PER™ Nuclear and Cytoplasmic Extraction Reagents Kit (Thermo Scientific, No. 78835) in accordance with the manufacturer's protocol. α‐tubulin and histone H3 served as internal controls for cytoplasmic and nuclear proteins, respectively. The concentrations of extracted proteins were measured by the BCA kit. Then proteins were denatured by heating at 99°C for 10 min, followed by separation using 10% SDS‐PAGE and transferred onto PVDF membranes. PVDF membranes were blocked using 5% bovine serum albumin for 2 h, followed by incubation with primary antibodies at 4°C overnight. Finally, they were incubated with secondary antibodies for 2 h. The antibodies used were listed as follows: rabbit Lyn (1:1000, Cell Signaling, 2732), rabbit *p*‐Lyn (1:1000, Cell Signaling, 70926), rabbit hexokinase 1 (HK1) (1:1000, Cell Signaling, 2024), rabbit triosephosphate isomerase 1 (TPI1) (1:1000, Abcam, ab196618), rabbit phosphoglycerate mutase 1 (PGAM1) (1:1000, Cell Signaling, 12098), rabbit 6‐phosphofructo‐2‐kinase/fructose‐2,6‐biphosphatase 3 (PFKFB3) (1:1000, Cell Signaling, 13123), rabbit IRF5 (1:1000, Cell Signaling, 96527), rabbit p‐IRF5 (1:1000, Invitrogen, PA5‐64760), rabbit SP1 (1:1000, Abcam, ab227383), rabbit PU.1 (1:1000, Abcam, ab227835), rabbit GAPDH (1:1000, Cell Signaling, 5174), rabbit α‐tubulin (1:1000, Cell Signaling, 2125), rabbit Histone H3 (1:2000, Cell Signaling, 4499), goat anti‐rabbit IgG (1:1000, Cell Signaling, 14708). Proteins were detected using enhanced chemiluminescence, and densitometry was performed using Image J software.

### 2.7 q‐PCR

Total RNA of spinal dorsal horn and cultured primary microglia were extracted using the TRIzol method. Then the collected RNA was reverse‐transcribed to cDNA with the reverse transcription kit according to the manufacturer's instructions. The polymerase chain reaction quantification was carried out using the SYBR Green kit by the QuantStudio 5 (Applied Biosystems). The cycle threshold (CT) values of target genes were collected and normalized to GAPDH, and the fold changes of gene expression was calculated with the 2^−ΔΔCT^ method. The primer sequences used in the experiments were listed in Table 1.

**TABLE 1:** Information for primer sequence of gene for *q*‐PCR.

Gene	Forward sequence	Reverse sequence
lyn	5′‐TGTGAGAGATCCAACGTCCA‐3′	5′‐AAACTGCCCTTGGCCATGTA‐3′
hk1	5′‐GCGAGGACAGGCTGTAGATG‐3′	5′‐CCGCATGGCATACAGATACTT‐3′
tpi1	5′‐TACAAAGTGACTAATGGGGC‐3′	5′‐TCGAAAACAACCTTCTCAGT‐3′
pgam1	5′‐ATGCTAAGCCATGACCAGTGAG‐3′	5′‐ATCACCACGCAGGTTACATTCG‐3′
pfkfb3	5′‐AGCCCGGATTACAAAGACTGC‐3′	5′‐GGTAGCTGGCTTCATAGCAAC‐3′
ym	5′‐AGAAGGGAGTTTCAAACCTGGT‐3′	5′‐CTCTTGCTGATGTGTGTAAGTGA‐3′
arg‐1	5′‐CTCCAAGCCAAAGTCCTTAGAG‐3′	5′‐AGGAGCTGTCATTAGGGACATC‐3′
il‐10	5′‐CCGAGATGCCTTCAGCAGAGT‐3′	5′‐GGAGTTCACATGCGCCTTGAT‐3′
tnf‐α	5′‐GTAGCCCACGTCGTAGCAAA‐3′	5′‐ACAAGGTACAACCCATCGGC‐3′
il‐1β	5′‐AGAGCCCATCCTCTGTGACT‐3′	5′‐GCTCATATGGGTCCGACAGC‐3′
il‐6	5′‐AACGATGATGCACTTGCAGA‐3′	5′‐TCTCTCTGAAGGACTCTGGCT‐3′
cyclin D1	5′‐GGAGCAGAAGTGCGAAGA‐3′	5′‐GGGCCGGATAGAGTTGTC‐3′
gapdh	5′‐TGCCACTCAGAAGACTGTGG‐3’	5′‐GGATGCAGGGATGATGTTCT‐3′

### 2.8 Immunofluorescence

Mice were euthanized and perfused with sterile saline and 4% paraformaldehyde. L4‐5 spinal cord of mice was quickly removed and post‐fixed in 4% paraformaldehyde for 1 day, and then moved into the 30% sucrose solution for dehydration. Next, tissue transverse frozen sections (20 μm thick) were cut using freezing microtome (Leica) and were incubated with 5% goat serum for 2 h at room temperature. Spinal cord sections were then incubated with primary antibodies overnight at 4°C. Before incubation with secondary antibodies, the sections were washed using PBS. After incubation with secondary antibodies for 2 h, digital images were captured with a high‐resolution CCD spot camera (fluorescent microscope) and then merged by Adobe Photoshop software. The mean fluorescence intensity was measured by Image J software. The antibodies used were listed as follows: rabbit Lyn (1:500, Abcam, ab137338), rabbit cyclin D1 (1:200, Abcam, ab16663), mouse IBA‐1 (1:100, Abcam, ab283319), goat anti‐rabbit IgG (1:500, Abcam, ab150083), goat anti‐mouse IgG (1:500, Abcam, ab150113).

### 2.9 Microdialysis

Samples from spinal dorsal horn of mice were collected by microdialysis to measure the levels of lactate, anti‐inflammatory phenotype markers or proinflammatory phenotype markers in spinal microenvironment. Mice were anaesthetised and placed on the stereotaxic instrument. A skin incision was carried out in the middle waist, and the vertebral plate of L4‐5 segment was removed to expose the spinal cord. The dialysis needles was then inserted into spinal dorsal horn. The dialysis tube was kept in the same plane with the spinal cord and fixed on microdialysis pump. Dialysate was pumped in the system for 2 h and then samples were collected for subsequent measurements.

### 2.10 Lactate measurement

Lactate concentration in spinal dorsal horn was tested by ELISA using the Lactate Assay Kit (Sigma, No. MAK064). Microenvironmental samples of spinal dorsal horn was acquired by microdialysis. By manufacturer's protocol, standards and samples were prepared in the sample diluent and six standards concentration gradients were established. Briefly, 50 μL of Detection A Working Solution was added immediately and incubated for 1 h at 37°C. Being aspirated and washed, each well added 100 μL of Detection B Working Solution, followed by incubation for 45 min at 37°C. After aspirating and washing again, each well added 90 μL of Substrate Solution, followed by incubation for 20 min in dark at 37°C. Then, 50 μL of Stop Solution was added to each well. Finally, the optical density of each well was determined simultaneously using PlateReader AF2000 (Eppendorf) at 570 nm.

### 2.11 Chromatin immunoprecipitation (ChIP)‐PCR assays

ChIP‐PCR assay was conducted to evaluate the binding of SP1 and PU.1 to glycolytic genes promoters using a ChIP Assay kit (Sigma, No. 17‐295) following the manufacturer's protocol. Samples from L4‐5 spinal dorsal horn and cultured primary microglia were sonicated and then cross‐linked with 1% formaldehyde at 37°C for 10 min, which was terminated by 125 mM glycine. After centrifugation, the acquired pellet was lysed by SDS lysis buffer with a protease inhibitor cocktail. Sonication conditions to the lysed sample were tested to yield DNA fragments averaging 600–800 bp as assessed by agarose gel electrophoresis. The input samples were collected as positive controls. After pre‐cleaned with protein G agarose, the samples were subjected to immunoprecipitation at 4°C overnight with 5 μL rabbit anti‐SP1 (Abcam, ab227383) and rabbit anti‐PU.1 (Abcam, ab227835). The precipitated protein‐DNA complexes were eluted, purified and subjected to *q*‐PCR for amplification of the promoter fragments of HK1, TPI1, PGAM1 and PFKFB3. The primer sequences of promoter fragments used in the experiments were listed in Table 2.

**TABLE 2:** Information for primer sequences of gene promoters for ChIP.

Promoter	Forward sequence	Reverse sequence
hk1	5′‐GCAAATGGTCCAGGCAGGGC‐3′	5′‐AGAGTTGGTTCAGACAAGGG‐3′
tpi1	5′‐GTCTGGGATGGGGGGGAGG‐3′	5′‐CCTCTTTGTCCCAGGCTGCCA‐3′
pgam1	5′‐GTCAACGGATTGCAGCAAGCC‐3′	5′‐CTCAGGCAAGTTCTGCGGCCT‐3′
pfkfb3	5′‐TGGAGGGATGTTGCTTACAG‐3′	5′‐TTAATAGACTATTGTTTCCCCCC‐3′
il‐6	5′‐CTTCTGATGTCTTGTTTAAACA‐3′	5′‐ATTAGGAGTCAACTCTCTAATT‐3′
ifn‐α	5′‐CCCCTTGCCAGCAATCCATTCC‐3′	5′‐GGTGAGTGTTGCCAAAGCTTCT‐3′

### 2.12 Statistical analysis

Statistical analyses were carried out using the IBM SPSS Statistics 23.0, data were presented as mean ± SEM. Two‐way repeated‐measure anova was used to compare mechanical withdrawal thresholds among different groups at different time points. The differences among groups were compared by one‐way anova analysis of variance followed by Dunnett's *t*‐test. A value of *p* < 0.05 was considered to be statistically significant.

## 3 RESULTS

### 3.1 GEO database reanalysis showed that Lyn might be associated with neuropathic pain

**FIGURE 1:**
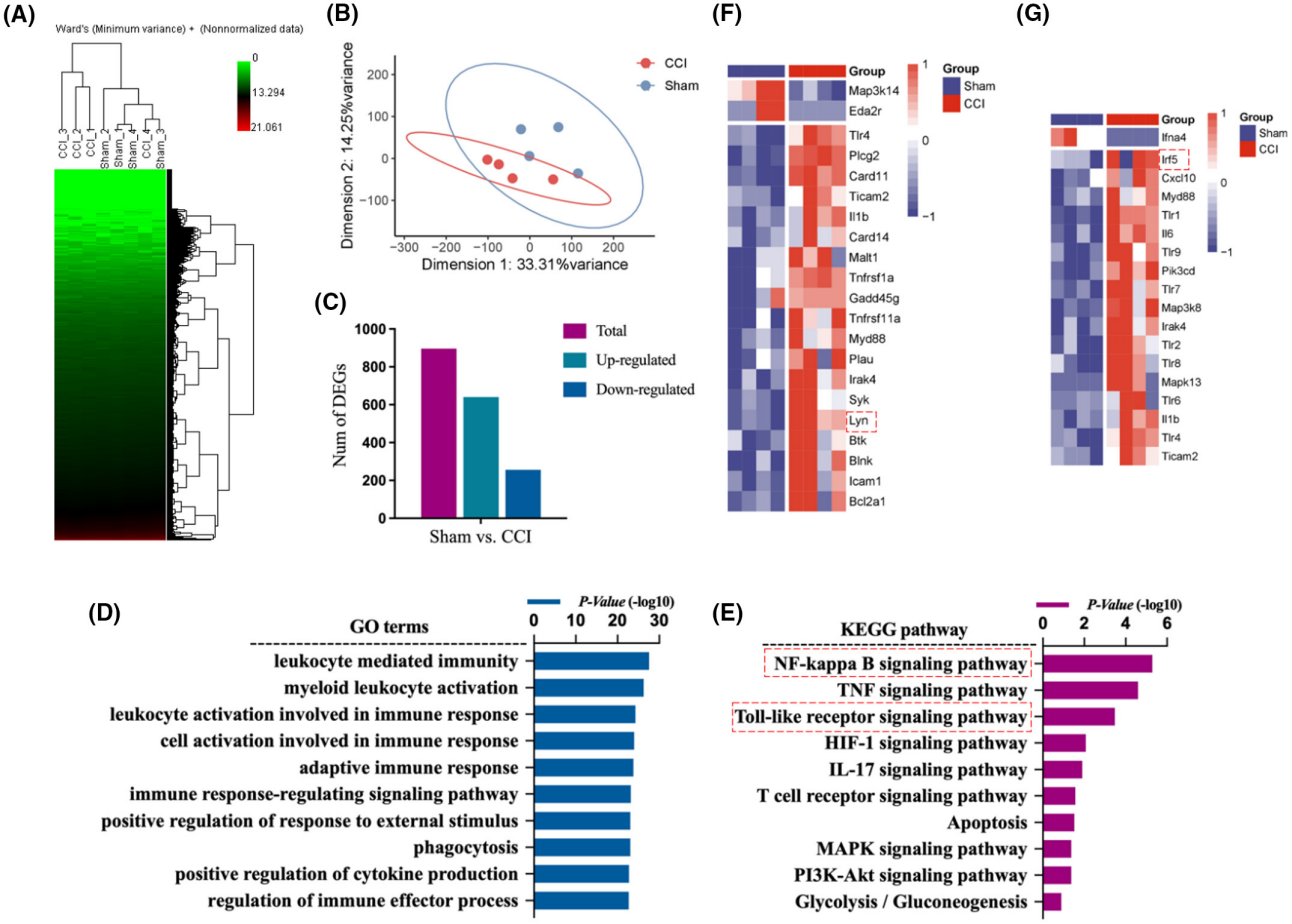
GEO database reanalysis showed that Lyn might be associated with neuropathic pain. (A) HCA plot of CCI and sham mice in GSE186237 datasets in GEO database; (B) PCA plot of CCI and sham mice; (C) bar graph presented the number of DEGs; (D) the GO analysis results; (E) the KEGG pathway enrichment results; (F) heatmap that showed the DEGs contained in these pathways including Lyn; (G) heatmap that showed the DEGs contained in these pathways including IRF5.

**FIGURE 2:**
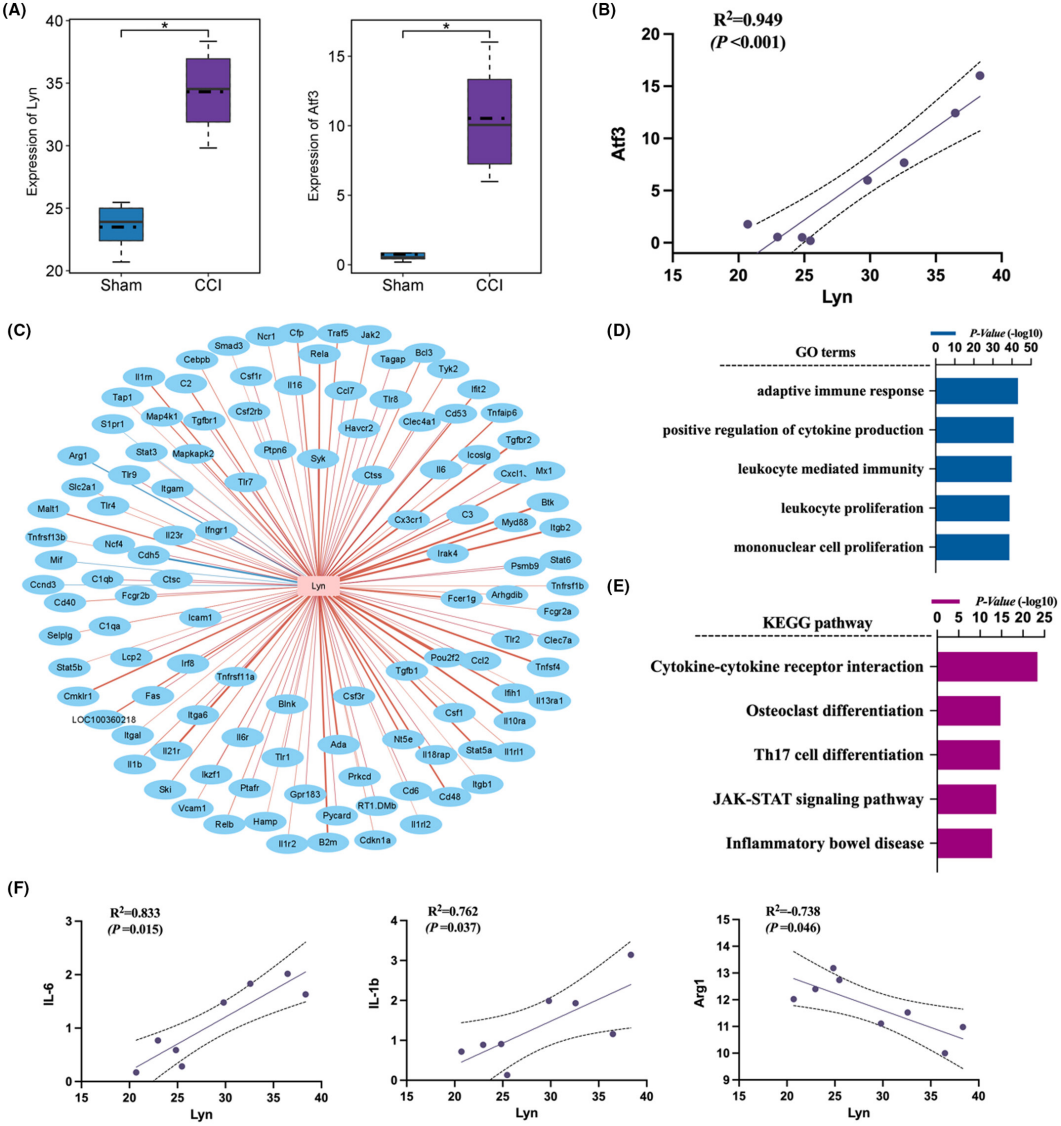
GEO database reanalysis showed the correlation between Lyn and inflammatory factors in neuropathic pain. (A) The expression levels of gene Lyn and Atf3 (a marker of nerve injury); (B) the correlation between Lyn and Atf3; (C) Lyn‐based co‐expression network analysis of the immune‐related transcriptome. The purple nodes represent Lyn gene, and the pink nodes represent immune‐related gene. The edges in red indicates upregulation, and blue indicates downregulation; (D) the GO analysis results of these co‐expressed inflammatory factors; (E) the KEGG pathway enrichment results of these co‐expressed inflammatory factors; (F) the correlation between Lyn and proinflammatory factors (IL‐6 and IL‐1β), anti‐inflammatory factor Arg1.

We reanalyzed the results of GSE186237 datasets in GEO database first. The HCA plot (Figure 1A) and PCA plot (Figure 1B) showed isolated views of the gene expression between different groups and the bar graph presented the number of DEGs (Figure 1C). The GO analysis results showed several immune‐related functions (Figure 1D) while the pathway enrichment results showed that the effects of these DEGs were mainly related to NF‐κB signalling pathway, TNF signalling pathway and Toll‐like receptor signalling pathway (Figure 1E). To further elucidate pain‐related mechanisms, we used heatmap to show the DEGs contained in these pathways, mainly including Lyn and IRF5 (Figure 1F,G). We further examined the expression levels of gene Lyn and Atf3 (a marker of nerve injury), and they showed a strong positive correlation (Figure 2A,B). We also examined the co‐expression relationship between Lyn gene and immune regulation related genes, among which some proinflammatory factors (IL‐6 and IL‐1β) were positively correlated with Lyn gene expression, while the anti‐inflammatory factor Arg1 expression was significantly negatively correlated (Figure 2C,F). The enrichment results of these co‐expressed inflammatory factors also suggest that neuroinflammation caused by CCI is closely related to Lyn gene, mainly related to pathways like cytokine‐cytokine receptor interaction, osteoclast differentiation and Th17 cell differentiation (Figure 2D,E).

### 3.2 CCI led to pain hyperalgesia and upregulation of Lyn expression in spinal dorsal horn

**FIGURE 3:**
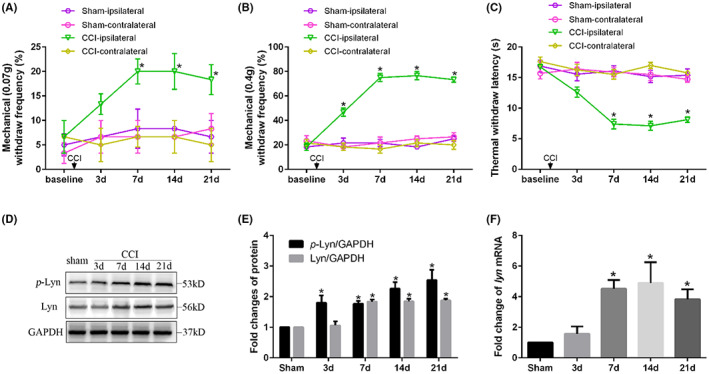
CCI led to pain hyperalgesia and upregulation of Lyn expression in spinal dorsal horn. (A) Mechanical withdraw frequencies to 0.07 g; (B) mechanical withdraw frequencies to 0.4 g; (C) thermal pain thresholds; (D) representative images of protein expressions of *p*‐Lyn and Lyn of mice; (E) statistical analysis of protein expressions of *p*‐Lyn and Lyn of mice; (F) mRNA level of Lyn of mice.

Then we measured the mechanical and thermal pain thresholds to confirm the successful establishment of CCI model of neuropathic pain. The mechanical withdraw frequencies to 0.07 g (*p* < 0.001) and 0.4 g (*p* < 0.001) both increased significantly on Day 7, 14 and 21 on the ipsilateral side of CCI mice, reached the peak on Day 7 and maintained more than 21 days compared with that of sham mice, suggesting that CCI induced pain allodynia and hyperalgesia (Figure 3A,B). Also, thermal withdraw latency decreased significantly on Days 7, 14 and 21 on the ipsilateral side of CCI mice compared with that of sham mice (*p* < 0.001) (Figure 3C), so we chose the mice 14 days after CCI as the stable neuropathic pain model in the following experiments. As a key member of the non‐receptor SFKs, Lyn tyrosine kinase regulates multiple processes in nerve injury and immune inflammatory signalling. *p*‐Lyn, the functional and activated type of Lyn, regulates protein activities by phosphorylating tyrosine residues of proteins. Western blot showed that the protein expression of both *p*‐Lyn (*p* = 0.004) and Lyn (*p* < 0.001) increased significantly on Day 7, 14 and 21 in CCI mice compared with that of sham mice in spinal dorsal horn (Figure 3D,E), and the increased mRNA level of Lyn in CCI mice was also confirmed by *q*‐PCR (*p* = 0.0002, Figure 3F), revealing the vital role of Lyn in spinal dorsal horn in the progression of neuropathic pain.

### 3.3 CCI‐induced Lyn upregulation mainly in microglia of the ipsilateral spinal dorsal horn

**FIGURE 4:**
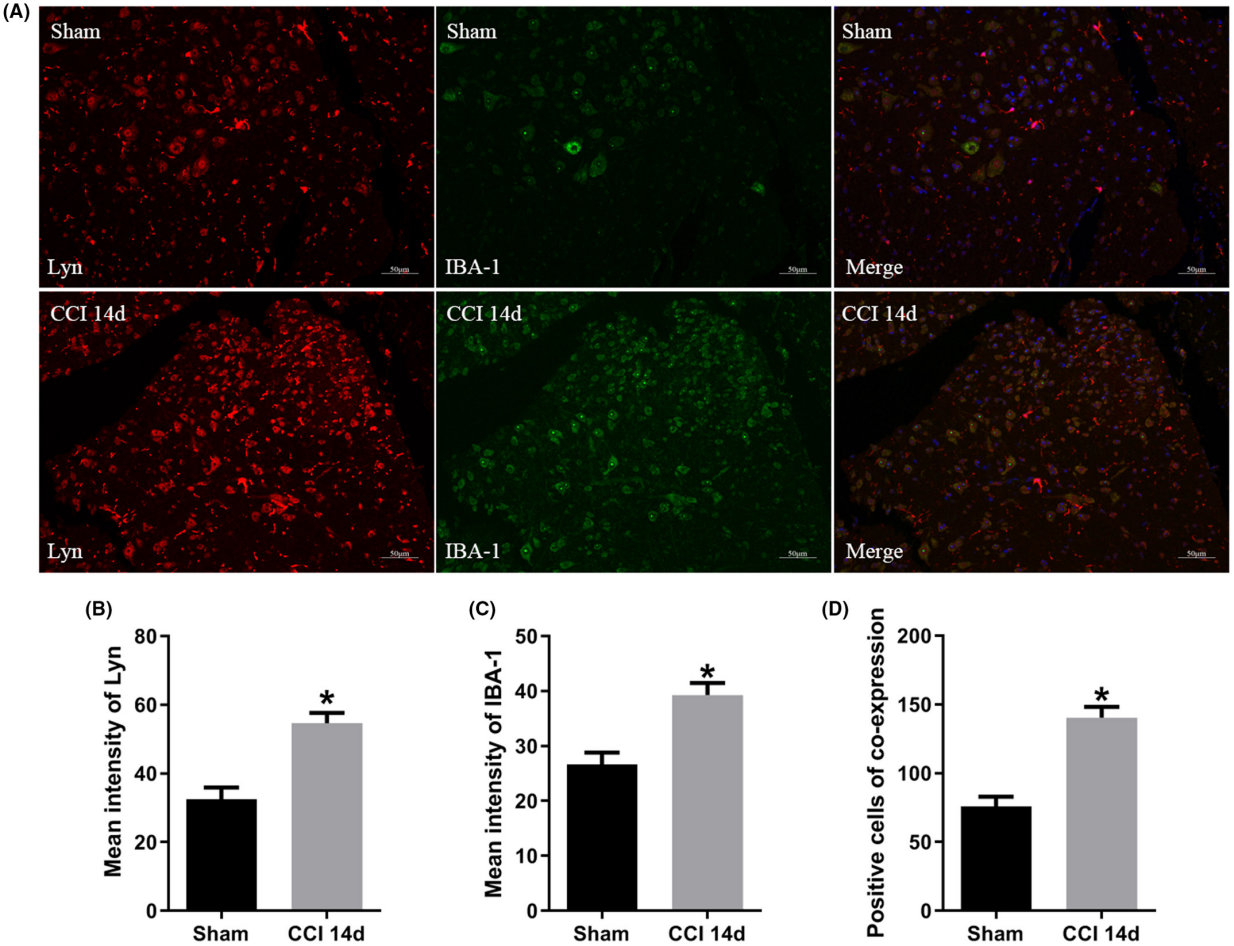
CCI‐induced Lyn upregulation mainly in microglia of the ipsilateral spinal dorsal horn. (A) Double staining of Lyn and microglia activation marker IBA‐1; (B) quantitative analysis of mean intensity of Lyn; (C) quantitative analysis of mean intensity of IBA‐1; (D) number of positive cell co‐expressing Lyn and IBA‐1.

Further we explored the cellular localization of Lyn in neuropathic pain. By double staining of Lyn and microglia activation marker IBA‐1, we confirmed that both Lyn (*p* = 0.008) and IBA‐1 (*p* = 0.015) increased on the ipsilateral spinal dorsal horn of CCI mice, and they also showed enhanced co‐expression (*p* = 0.004) compared with that of sham mice (Figure 4A), the quantitative analysis of mean intensity also confirmed the results (Figure 4B,C,D), suggesting that the microglia was activated and upregulated Lyn expression in the progression of neuropathic pain after CCI.

### 3.4 Lyn expression was also upregulated in cultured primary microglia under LPS or IFN‐γ stimulation

**FIGURE 5:**
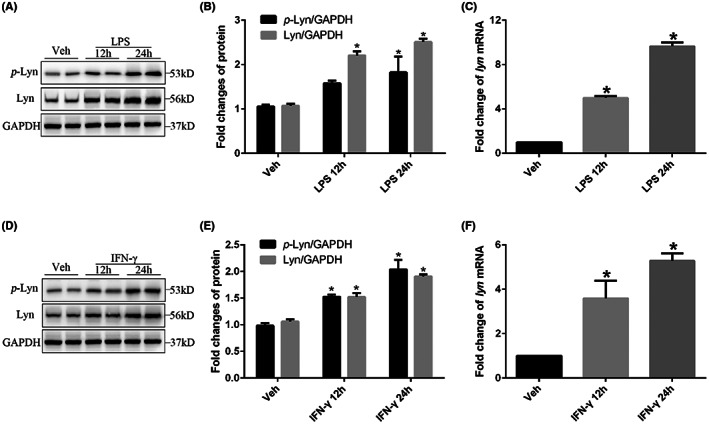
Lyn expression was also upregulated in cultured primary microglia under LPS or IFN‐γ stimulation. (A, D) Representative images of protein expressions of *p*‐Lyn and Lyn of cultured primary microglia under LPS stimulation; (B, E) statistical analysis of protein expressions of *p*‐Lyn and Lyn of cultured primary microglia; (C, F) mRNA level of Lyn of cultured primary microglia.

To further confirm the relationship of Lyn and microglia, we also observed the Lyn expression changes in cultured primary microglia. LPS or IFN‐γ were administrated to stimulate microglia activation. Western blot showed that the proteins expression of both *p*‐Lyn (*p* < 0.001) and Lyn (*p* < 0.001) increased significantly at 12 and 24 h after LPS administration compared with that of vehicle administration in cultured primary microglia (Figure 5A, B), and the increased mRNA level of Lyn after LPS administration was also confirmed by *q*‐PCR (*p* < 0.001, Figure 5C). Similarity, both increased protein expression (*p* < 0.001 for *p*‐Lyn and Lyn) and mRNA level (*p* < 0.001) of Lyn were confirmed at 12 and 24 h after IFN‐γ administration compared with that of vehicle administration in cultured primary microglia (Figure 5D–F), revealing the crucial relationship between Lyn and microglia activation.

### 3.5 Lyn inhibitor Bafetinib or siRNA‐lyn knockdown intrathecally alleviated pain hyperalgesia in CCI mice

**FIGURE 6:**
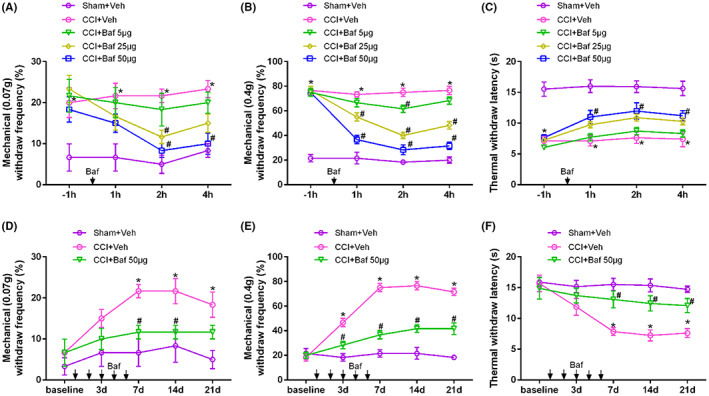
Lyn inhibitor Bafetinib intrathecally alleviated pain hyperalgesia in CCI mice. (A) Mechanical withdraw frequencies to 0.07 g after single intrathecal administration of different doses of Bafetinib on CCI mice; (B) mechanical withdraw frequencies to 0.4 g after single intrathecal administration of different doses of Bafetinib on CCI mice; (C) thermal pain thresholds after single intrathecal administration of different doses of Bafetinib on CCI mice; (D) mechanical withdraw frequencies to 0.07 g after continuous Bafetinib intrathecally on CCI mice; (E) mechanical withdraw frequencies to 0.4 g after continuous Bafetinib intrathecally on CCI mice; (F) Thermal pain thresholds after continuous Bafetinib intrathecally on CCI mice.

Considering the facilitation of microglia activation on neuropathic pain, we next explored the effects of Lyn inhibitor Bafetinib or siRNA‐lyn intrathecally on pain hyperalgesia in CCI mice. After single intrathecal administration of different doses of Bafetinib on CCI mice, we found that the mechanical withdraw frequencies to 0.07 g (*p* < 0.001) and 0.4 g (*p* < 0.001) both decreased at Hours 1, 2 and 4, reached the bottom at Hour 2 and maintained more than 4 h in a dose dependent manner compared with that of vehicle intrathecally on CCI mice (Figure 6A,B), suggesting that Bafetinib intrathecally alleviated pain allodynia and hyperalgesia in CCI mice. Also, thermal withdraw latency increased significantly at Hours 1, 2 and 4 after 50 μg Bafetinib intrathecally compared with that of vehicle intrathecally on CCI mice (*p* < 0.001, Figure 6C), so we chose the dose 50 μg of Bafetinib intrathecally to effectively inhibit Lyn function. Further, we tested whether continuous Bafetinib intrathecally after CCI could reverse the progression of pain hyperalgesia. After continuous Bafetinib intrathecally for 5 days after CCI, the mechanical withdraw frequencies to 0.07 g (*p* < 0.001) and 0.4 g (*p* < 0.001) both decreased significantly on Day 7, 14 (Figure 6D, E), while thermal withdraw latency increased significantly on Day 7, 14, 21 compared with that of vehicle intrathecally on CCI mice (*p* < 0.001, Figure 6F), suggesting that continuous Bafetinib intrathecally effectively reversed the progression of neuropathic pain.

**FIGURE 7:**
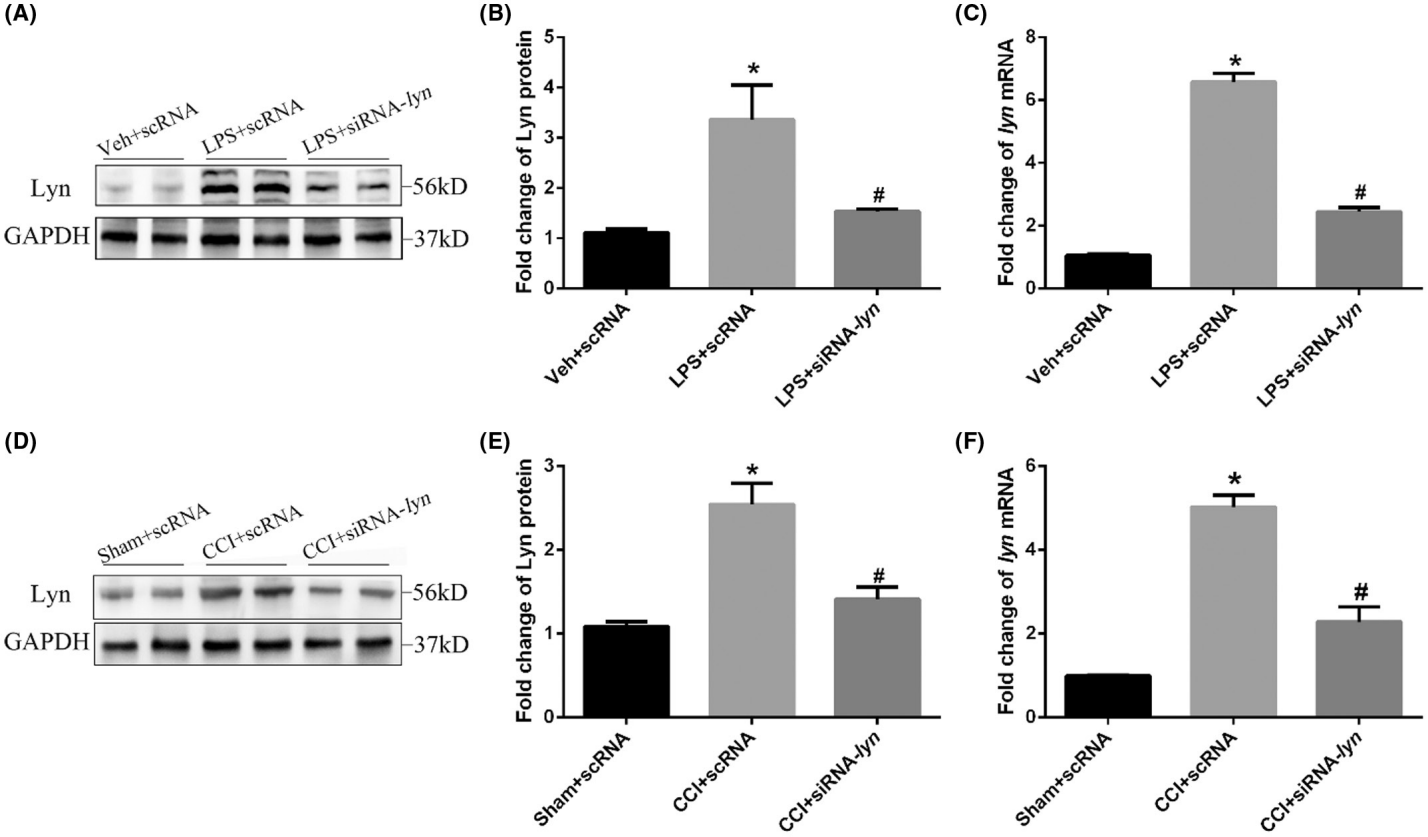
siRNA‐lyn knockdown efficiency. (A) Representative images of protein expression of Lyn in cultured primary microglia after siRNA‐lyn knockdown; (B) statistical analysis of protein expression of Lyn in cultured primary microglia after siRNA‐lyn knockdown; (C) mRNA level of Lyn in cultured primary microglia after siRNA‐lyn knockdown; (D) representative images of protein expression of Lyn of mice after siRNA‐lyn knockdown; (E) statistical analysis of protein expressions of Lyn of mice after siRNA‐lyn knockdown; (F) mRNA level of Lyn of mice after siRNA‐lyn knockdown.

**FIGURE 8:**
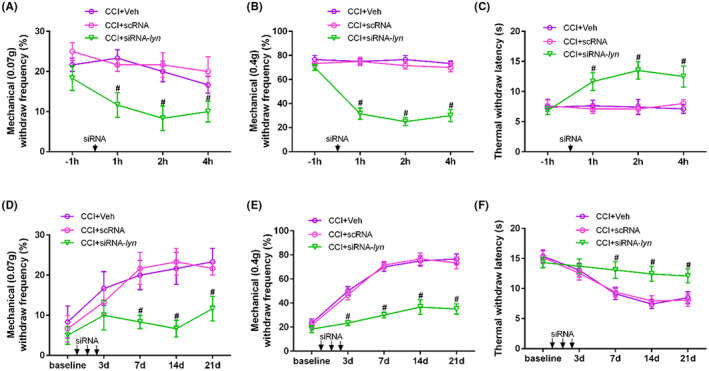
siRNA‐lyn knockdown intrathecally alleviated pain hyperalgesia in CCI mice. (A) Mechanical withdraw frequencies to 0.07 g after single siRNA‐lyn intrathecally on CCI mice; (B) mechanical withdraw frequencies to 0.4 g after single siRNA‐lyn intrathecally on CCI mice; (C) thermal pain thresholds after single siRNA‐lyn intrathecally on CCI mice; (D) mechanical withdraw frequencies to 0.07 g after continuous siRNA‐lyn intrathecally on CCI mice; (E) mechanical withdraw frequencies to 0.4 g after continuous siRNA‐lyn intrathecally on CCI mice; (F) thermal pain thresholds after continuous siRNA‐lyn intrathecally on CCI mice.

Also, siRNA targeting knockdown Lyn was also administrated intrathecally in CCI mice. We first tested the knockdown efficiency of siRNA in cultured primary microglia. The protein (*p* = 0.006) and mRNA levels (*p* < 0.001) of Lyn were significantly increased in LPS + scRNA microglia compared with that in veh + scRNA, while they were significantly decreased in LPS + siRNA‐lyn microglia compared with that in LPS + scRNA (*p* = 0.02 for protein and *p* < 0.001 for mRNA) (Figure 7A–C). Similarity, intrathecal administration of siRNA‐lyn in CCI mice also significantly decreased the protein (*p* < 0.001) and mRNA levels (*p* < 0.001) of Lyn compared with that in CCI + scRNA mice in spinal dorsal horn (Figure 7D,E,F), suggesting the efficient knockdown of Lyn both in cultured primary microglia and spinal dorsal horn of mice. After single intrathecal administration of siRNA‐lyn on CCI mice, the mechanical withdraw frequencies to 0.07 g (*p* < 0.001) and 0.4 g (*p* < 0.001) both decreased at Hours 1, 2 and 4, reached the bottom at hour 2 and maintained more than 4 h compared with that of scRNA intrathecally on CCI mice (Figure 8A,B), suggesting that siRNA‐lyn intrathecally alleviated pain allodynia and hyperalgesia in CCI mice. Also, thermal withdraw latency increased significantly at Hours 1, 2 and 4 after siRNA‐lyn intrathecally compared with that of scRNA intrathecally on CCI mice (*p* < 0.001, Figure 8C). After continuous siRNA‐lyn intrathecally for 3 days after CCI, the mechanical withdraw frequencies to 0.07 g (*p* < 0.001) and 0.4 g (*p* < 0.001) both decreased significantly on Day 7, 14, 21 (Figure 8D,E), while thermal withdraw latency increased significantly on Day 7, 14, 21 compared with that of scRNA intrathecally on CCI mice (*p* < 0.001, Figure 8F), suggesting that continuous siRNA targeting knockdown Lyn intrathecally effectively reversed the progression of neuropathic pain.

### 3.6 CCI‐induced glycolysis enhancement was suppressed by Bafetinib or Lyn siRNA intrathecally

**FIGURE 9:**
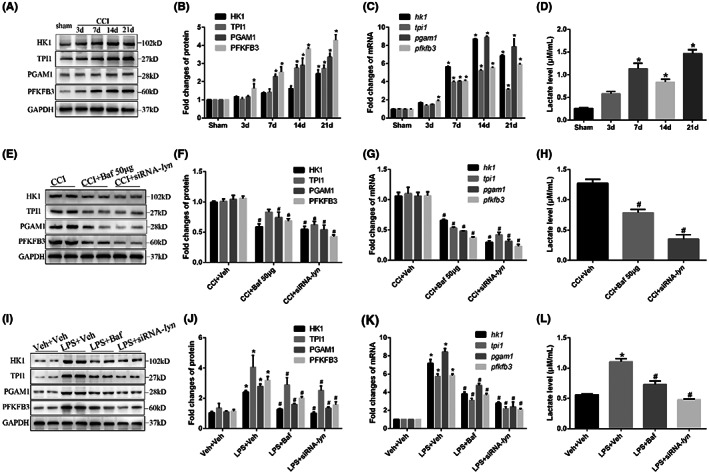
CCI‐induced glycolysis enhancement was suppressed by Bafetinib or siRNA‐lyn intrathecally. (A) Representative images of protein expressions of key enzymes in glycolysis (HK1, TPI1, PGAM1 and PFKFB3) in CCI mice; (B) statistical analysis of protein expressions of key enzymes in glycolysis in CCI mice; (C) mRNA levels of key enzymes in glycolysis in CCI mice; (D) lactate level in spinal dorsal horn of CCI mice by microdialysis; (E) representative images of protein expressions of key enzymes in glycolysis after Bafetinib and siRNA‐lyn intrathecally in CCI mice; (F) statistical analysis of protein expressions of key enzymes in glycolysis after Bafetinib and siRNA‐lyn intrathecally in CCI mice; (G) mRNA levels of key enzymes in glycolysis after Bafetinib and siRNA‐lyn intrathecally in CCI mice; (H) lactate level after Bafetinib and siRNA‐lyn intrathecally in spinal dorsal horn of CCI mice by microdialysis; (I) representative images of protein expressions of key enzymes in glycolysis after Bafetinib and siRNA‐lyn administration in cultured primary microglia; (J) statistical analysis of protein expressions of key enzymes in glycolysis after Bafetinib and siRNA‐lyn administration in cultured primary microglia; (K) mRNA levels of key enzymes in glycolysis after Bafetinib and siRNA‐lyn administration in cultured primary microglia; (L) lactate level in cultured primary microglia after Bafetinib and siRNA‐lyn administration.

As is confirmed that glycolysis is closely related to neuroinflammation in spinal dorsal horn in neuropathic pain, we next tested whether Lyn regulated microglia glycolysis. The key enzymes in glycolysis (HK1, TPI1, PGAM1 and PFKFB3) were used to evaluate glycolysis activities in microglia during neuropathic pain. Western blot and *q*‐PCR both confirmed that the proteins expression (*p* < 0.001) and mRNA levels (*p* < 0.001) of the key enzymes increased significantly on Day 7, 14, 21 after CCI in a time dependent manner compared with that in sham mice (Figure 9A–C), and the lactate level in spinal dorsal horn by microdialysis also elevated on Days 7, 14 and 21 compared with that in sham mice (*p* < 0.001, Figure 9D), revealing that CCI induced glycolysis enhancement in spinal dorsal horn. However, inhibiting Lyn by Bafetinib (*p* < 0.001) or siRNA‐lyn (*p* < 0.001) intrathecally in CCI mice both decreased the proteins expression and mRNA levels of the key enzymes (Figure 9E–G), and decreased lactate level in spinal dorsal horn compared with that in CCI + veh mice (*p* = 0.003 and *p* < 0.001, Figure 9H). Similarity, LPS significantly increased the proteins expression and mRNA levels of the key enzymes (*p* < 0.001, Figure 9I–K), and increased lactate level in cultured primary microglia in vitro (*p* < 0.001, Figure 9L), but these effects were all weakened by Bafetinib (*p* < 0.001) and siRNA‐lyn (*p* < 0.001) administration. These results all implied that Lyn enhanced the glycolysis activities in spinal dorsal horn in the progression of neuropathic pain.

### 3.7 Lyn facilitated the nuclear translocation of IRF5

**FIGURE 10:**
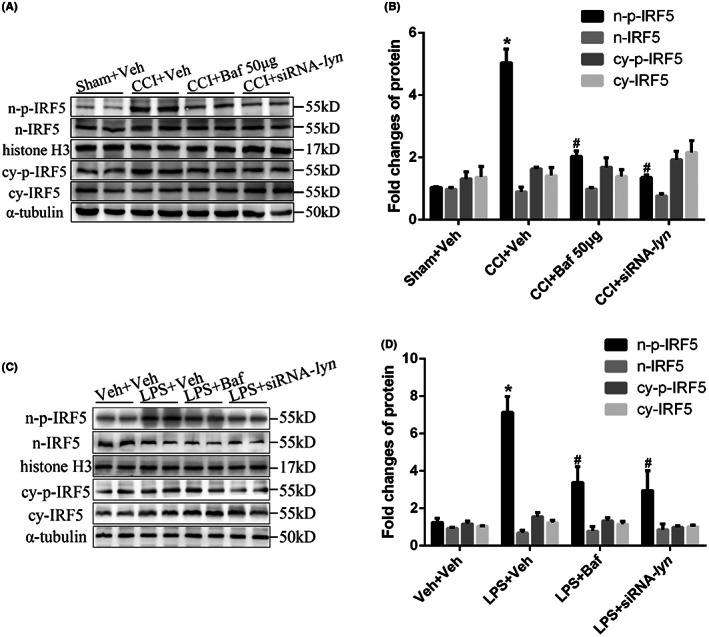
Lyn facilitated the nuclear translocation of IRF5. (A) Representative images of protein expressions of n‐p‐IRF5, n‐IRF5, cy‐p‐IRF5, cy‐IRF5 after Bafetinib and siRNA‐lyn intrathecally in CCI mice; (B) statistical analysis of protein expressions of n‐p‐IRF5, n‐IRF5, cy‐p‐IRF5, cy‐IRF5 after Bafetinib and siRNA‐lyn intrathecally in CCI mice; (C) representative images of protein expressions of n‐p‐IRF5, n‐IRF5, cy‐p‐IRF5, cy‐IRF5 after Bafetinib and siRNA‐lyn administration in cultured primary microglia; (D) statistical analysis of protein expressions of n‐p‐IRF5, n‐IRF5, cy‐p‐IRF5, cy‐IRF5 after Bafetinib and siRNA‐lyn administration in cultured primary microglia.

Given that IRF5 regulates LPS‐initiated cytokine secretion in human myeloid‐derived cells through regulating gene transcription in glycolysis, we hypothesized that IRF5 would be necessary for CCI‐induced glycolysis enhancement in microglia. The nuclear phosphorylated IRF5 (n‐p‐IRF5) derived from nuclear translocation mainly acquires the ability to regulate gene transcription in glycolysis, so we measured the cytoplasmic and nuclear IRF5 (cy‐IRF5 and n‐IRF5) levels to evaluate whether Lyn regulate the nuclear translocation of IRF5. In vivo, n‐p‐IRF5 level was significantly higher in CCI mice compared with that in sham mice (*p* < 0.001), while the change was reversed by Bafetinib (*p* < 0.001) or siRNA‐lyn (*p* < 0.001) intrathecally in CCI mice. The n‐IRF5, cy‐p‐IRF5, cy‐IRF5 levels showed no significant differences in sham and CCI mice with or without Bafetinib or siRNA‐lyn intrathecally (all *p* > 0.05, Figure 10A,B). In vitro, LPS significantly elevated n‐p‐IRF5 level in cultured primary microglia (*p* < 0.001), while it was reversed by Bafetinib (*p* < 0.001) or siRNA‐lyn (*p* < 0.001) administration. However, LPS, Bafetinib or siRNA‐lyn administration all did not change the n‐IRF5, cy‐p‐IRF5, cy‐IRF5 levels in cultured primary microglia (all *p* > 0.05, Figure 10C,D). The vivo and vitro results confirmed that Lyn facilitated the nuclear translocation of IRF5 in microglia in spinal dorsal horn in the progression of neuropathic pain.

### 3.8 IRF5 promoted the binding of transcription factors SP1, PU.1 to glycolytic gene promoters

**FIGURE 11:**
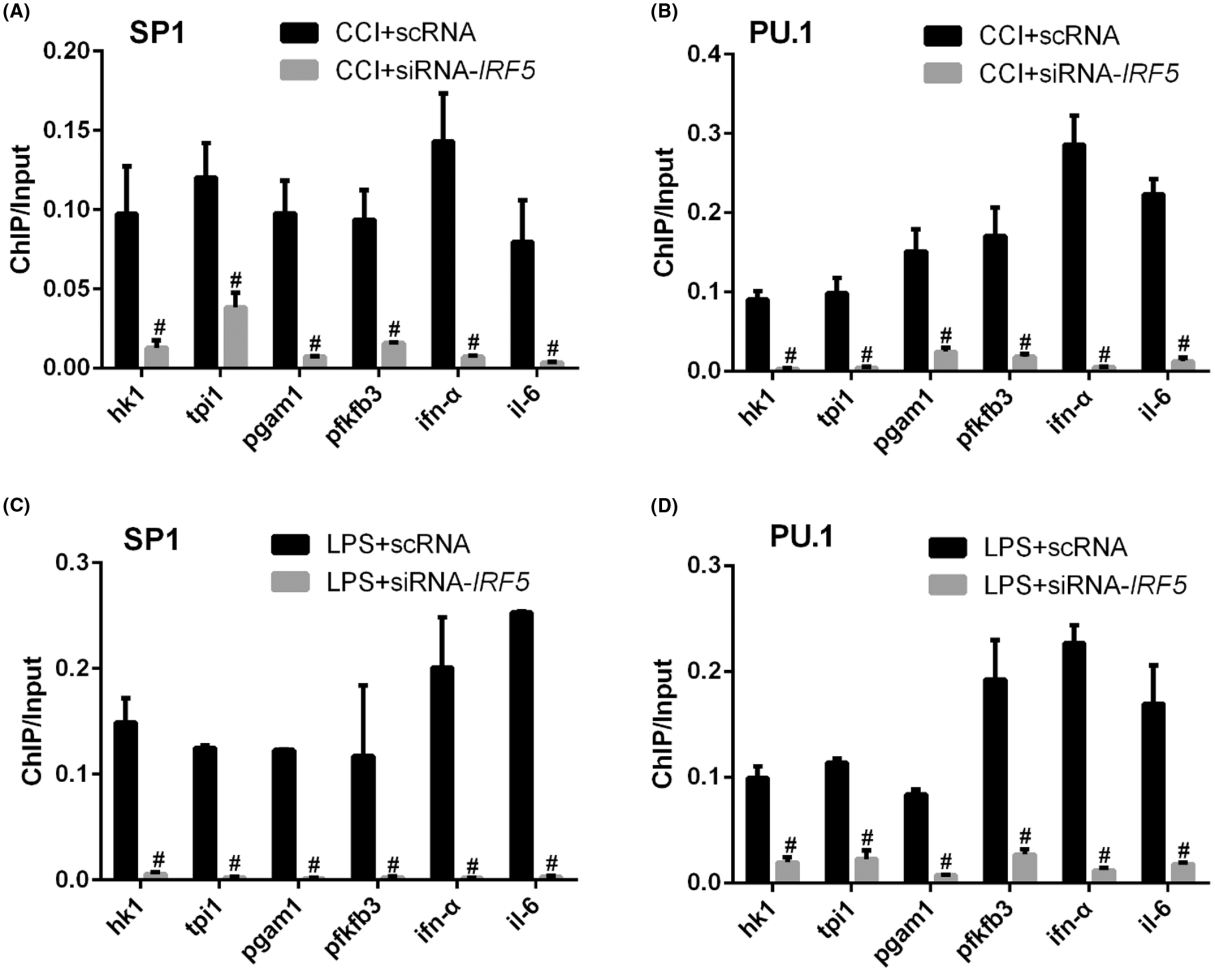
IRF5 promoted the binding of transcription factors SP1, PU.1 to glycolytic gene promoters. (A) The binding of SP1 to glycolytic gene promoters HK1, TPI1, PGAM1, PFKFB3 and positive controls IL‐6, IFN‐α after siRNA‐IRF5 intrathecally in CCI mice by ChIP; (B) the binding of PU.1 to glycolytic gene promoters and positive controls after siRNA‐IRF5 intrathecally in CCI mice by ChIP; (C) the binding of SP1 to glycolytic gene promoters and positive controls after siRNA‐IRF5 administration in cultured primary microglia by ChIP; (D) the binding of PU.1 to glycolytic gene promoters and positive controls after siRNA‐IRF5 administration in cultured primary microglia by ChIP.

Nuclear IRF5 is both a transcription factor and regulator of downstream SP1, PU.1, which can directly interact with gene promoters to initiate transcription. We therefore sought to dissect whether IRF5 could regulate CCI‐induced glycolytic outcomes by directly promoting the binding of transcription factors SP1, PU.1 to the glycolytic genes promoters. CHIP was used to evaluate the binding of SP1, PU.1 to promoters of HK1, TPI1, PGAM1 and PFKFB3. IL‐6 and IFN‐α promoters were included as positive controls as they were admittedly binding to SP1 and PU.1. IRF5 knockdown with siRNA‐IRF5 intrathecally significantly decreased the binding of both SP1 and PU.1 to the promoters of HK1, TPI1, PGAM1 and PFKFB3 (*p* < 0.001, Figure 11A,B). Also, IRF5 knockdown with siRNA‐IRF5 administration in cultured primary microglia significantly decreased the binding of both SP1 and PU.1 to the promoters of HK1, TPI1, PGAM1 and PFKFB3 (*p* < 0.001, Figure 11C,D). The vivo and vitro results revealing that IRF5 promoted the binding of transcription factors SP1, PU.1 to glycolytic gene promoters in microglia in spinal dorsal horn.

### 3.9 Enhanced glycolysis facilitated proliferation and proinflammatory phenotype transition of microglia

**FIGURE 12:**
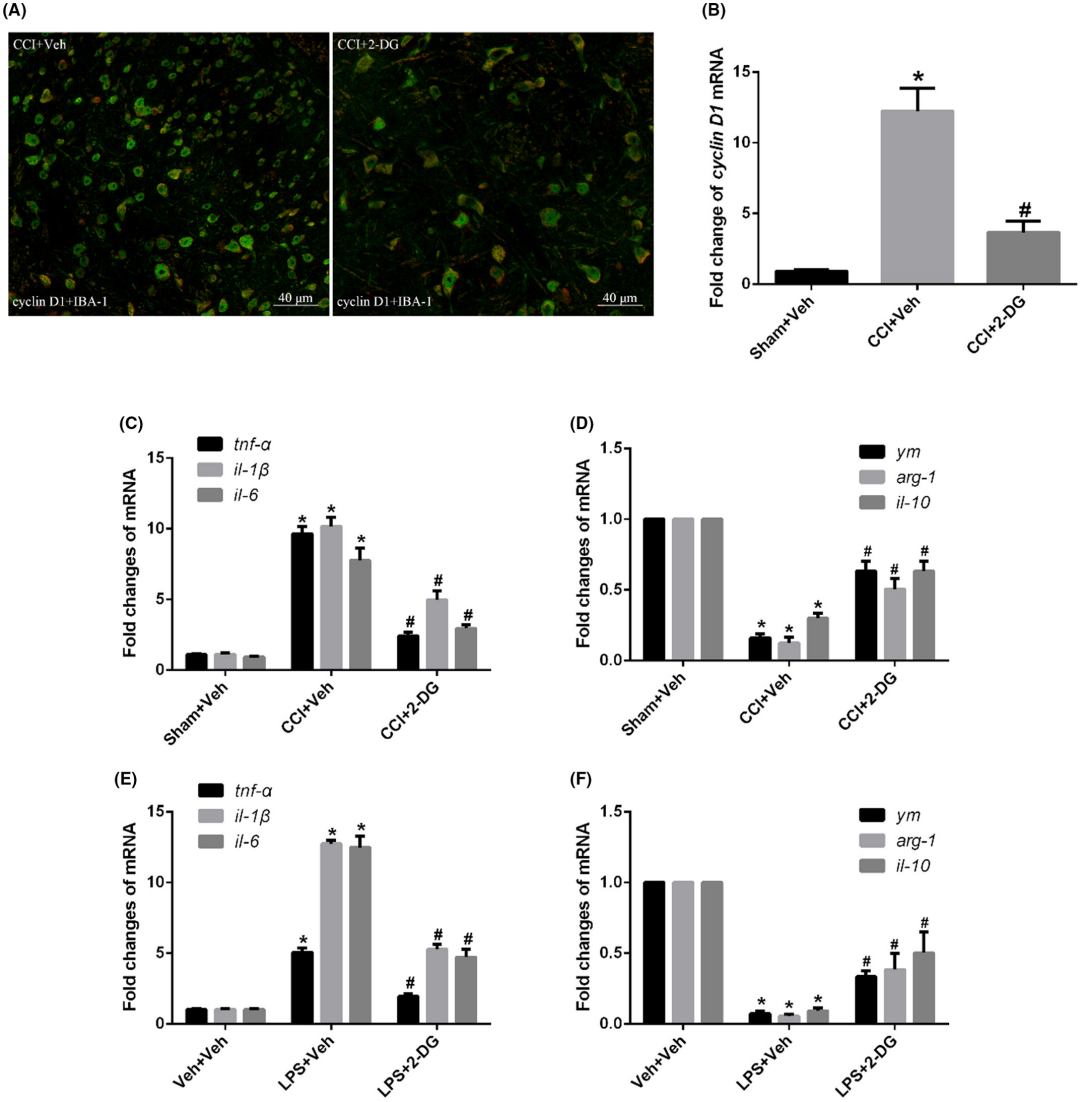
Enhanced glycolysis facilitated proliferation and proinflammatory phenotype transition of microglia. (A) Double staining of cyclin D1 and IBA‐1 after glycolysis inhibitor 2‐DG intrathecally; (B) mRNA levels of cyclin D1 after 2‐DG intrathecally in CCI mice; (C) mRNA levels of pro‐inflammatory phenotype markers (tnf‐α, il‐1β, il‐6) after 2‐DG intrathecally in CCI mice; (D) mRNA levels of anti‐inflammatory phenotype markers (ym, arg‐1, il‐10) after 2‐DG intrathecally in CCI mice; (E) mRNA levels of pro‐inflammatory phenotype markers after 2‐DG administration in cultured primary microglia; (F) mRNA levels of anti‐inflammatory phenotype markers after 2‐DG administration in cultured primary microglia.

Given that microglia in proinflammatory phenotype contributes vitally to neuropathic pain, we further explored the effects of CCI‐induced enhanced glycolysis on proliferation and proinflammatory phenotype transition of microglia. As a key regulator of the G1 phase in cell cycle, cyclin D1 was regarded as the marker of cell proliferation. Immunofluorescence showed that the co‐expression of cyclin D1 and IBA‐1 increased in the spinal dorsal horn of CCI mice (Figure 12A), *q*‐PCR showed that the mRNA expression of cyclin D1 also increased in CCI mice compared with that in sham mice (*p* < 0.001, Figure 12B), but these changes were alleviated by glycolysis inhibitor 2‐DG intrathecally (*p* = 0.0004), suggesting that CCI‐induced enhanced glycolysis facilitated proliferation of microglia. Next we used *q*‐PCR to measure the mRNA levels of anti‐inflammatory phenotype markers (ym, arg‐1, il‐10) and pro‐inflammatory phenotype markers (tnf‐α, il‐1β, il‐6). The mRNA levels of ym, arg‐1, il‐10 were significantly lower (*p* < 0.001) while the mRNA levels of tnf‐α, il‐1β, il‐6 were significantly higher (*p* < 0.001) in CCI mice compared with that in sham mice, but these changes were alleviated by glycolysis inhibitor 2‐DG intrathecally in vivo (*p* < 0.001, Figure 12C,D). Similarity, LPS administration significantly reduced the mRNA levels of ym, arg‐1, il‐10 (*p* < 0.001) and elevated the mRNA levels of tnf‐α, il‐1β, il‐6 (*p* < 0.001) in cultured primary microglia, while they were alleviated by 2‐DG administration in vivo (*p* < 0.001, Figure 12E,F), confirming that CCI‐induced enhanced glycolysis facilitated the pro‐inflammatory phenotype transition of microglia.

## 4 DISCUSSION

Glycometabolism reprogramming has been confirmed to be a significant inducer of pro‐inflammatory phenotype transition of microglia to affect synaptic remodelling in the progression of neuropathic pain.^17^ Lyn is reported to modify the tyrosine residues of proteins to regulate the key enzymes in glycometabolism reprogramming, but the specific mechanism remains unclear. Here we identified that Lyn mainly located in microglia and was upregulated in the ipsilateral spinal dorsal horn of CCI mice and cultured primary microglia. Lyn inhibitor Bafetinib or siRNA‐lyn knockdown intrathecally alleviated pain hyperalgesia in CCI mice. We also confirmed that Lyn facilitated the nuclear translocation of IRF5 and thereafter promoted the binding of transcription factors SP1, PU.1 to glycolytic gene promoters, resulting in glycolysis enhancement after CCI. The enhanced glycolysis facilitated proliferation and pro‐inflammatory phenotype transition of microglia to induce synaptic remodelling in the progression of neuropathic pain.

Neuroinflammation is a common feature of most nervous dysfunctions like multiple sclerosis, Alzheimer's disease or pathological pain.^18^ Various types of cells are involved in the development of neuroinflammation in central nervous system, especially microglia and astrocyte. Acting as immune cells in central nervous system, microglia can activate inflammasome, NF‐κB and other inflammatory signalling pathways.^19^ In the resting state, microglia sense the changes of microenvironment around synapses and mainly relies on oxidative phosphorylation for glucose metabolism. Facing nocuous factors, microglia activates a series of stress responses to shift into activated state, and takes in more glucose for the enhanced glycolysis.^20^, ^21^ Recent studies have implied that multiple mechanisms are involved in glycometabolism shift to promote the transcription of genes associated with pro‐inflammatory factors, including the accumulation of advanced glycation end products (AGEs) derived from proteins or lipids in microglia by binding to AGEs receptors to facilitate the combination of promoters and pro‐inflammatory genes.^22^ Abnormal activation of microglia can also accelerate autophagy and apoptosis of neurons during neurodegeneration. Acute short‐term exposure to β amyloid of microglia induced the shift from oxidative phosphorylation to glycolysis through the mammalian target of rapamycin/hypoxia inducible factor‐1α (mTOR/HIF‐1α) pathway. However, chronic long‐term exposure to β amyloid of microglia attenuated both glycolysis and oxidative phosphorylation, and reduced the responsiveness of microglia to noxious stimulation.^23^, ^24^ In Alzheimer's disease mice, exogenous IFN‐γ improved the neurological symptoms by attenuating the stimulation of β amyloid to microglia through promoting glycolysis by activating mTOR pathway.^17^ Further research found that mice with associated genes knockout in Alzheimer's disease showed decreased mTOR pathway activity, impaired glycolysis function and increased neuronal autophagy, especially the triggering receptor expressed on myeloid cells‐2 (TREM‐2) gene.^25^ Multiple sclerosis patients are mainly characterized by white matter inflammatory demyelinating in central nervous system caused by autoimmune injury, and these patients are also accompanied by obvious glycolysis enhancement in activated microglia.^26^ These metabolic changes may be the potential basis in treating multiple sclerosis by targeting glycolysis and mitochondrial metabolism in microglia.

With the stimulation of CCI, glucometabolic reprogramming in microglia is mainly characterized by increased glycolysis and lactate production, enhanced glutamine hydrolysis and pentose phosphate pathway and weakened TCA cycle.^27^ These metabolic transformation leads to the accumulation of various intermediates like phosphoenolpyruvic acid (PEP), succinate, citric acid, methylene succinic acid, α‐ketoglutaric acid, lactate and 2‐hydroxyglutaric acid. These intermediates can affect the acid–base balance of cell microenvironment, promote transcription of pro‐inflammatory factors and activate inflammatory signalling pathways to change the inflammatory phenotypes of peripheral immune cells and central microglia.^28^ By activating microglia in CCI mice, the intracellular glycolysis is enhanced and PEP is accumulated in cells with the catalysing of enolase‐1, which interferes with Ca^2+^ signalling and promotes the formation of inflammatory waterfalls. PEP accumulation by glycolysis can inhibit the calcium channels in endoplasmic reticulum, resulting in the obstacle for calcium in entering into the calcium reservoir in endoplasmic reticulum. These changes all result in significantly increase of calcium concentration in cytoplasm, further activating inflammatory pathways such as NF‐κB to maintain the activation state of microglia and promote the transcription of pro‐inflammatory factors. These changes in microglia facilitate synaptic remodelling vitally in neuropathic pain.^29^, ^30^ Also, we observed the pro‐inflammatory responses of microglia suffering from noxious stimulation in vivo and in vitro, which was alleviated by inhibiting glycolysis, revealing the close connection between glycolysis and pro‐inflammatory phenotype of microglia.

However, the regulatory mechanism of glycolysis in neuropathic pain still remains vague. We focused on the SFKs for their epigenetic modification function in gene transcription of key enzymes in glycolysis. In SFKs, Lyn participates in immune inflammatory responses, cell proliferation, differentiation, apoptosis, migration and metabolism. In the regulation of B cell receptor signalling, activated Lyn phosphorylates tyrosine residues within immunoreceptor tyrosine‐based activation or inhibition motifs (ITAMs or ITIMs) to recruit regulator proteins.^31^ Recent studies have shown that Lyn inhibits innate immune signalling along the TLR‐MyD88 axis in dendritic cells and B cells. Specifically, Lyn‐deficient dendritic cells exhibit hyper‐responsiveness to inflammatory inducers, suggesting the strong influences of Lyn on immune and inflammatory signalling pathways.^32^ We also observed the increased expression of Lyn in microglia of spinal dorsal horn after CCI and observed the crucial impact of Lyn on allodynia and hyperalgesia in neuropathic pain by Bafetinib or siRNA‐lyn intrathecally. We confirmed that Lyn strengthened microglia glycolysis after CCI by measuring key enzyme expressions in glycolysis and lactate level, which was closely related to neuropathic pain. To further explore the mechanism of how Lyn affects glycolysis, we focused on the transcription factor IRF5, which formed a regulatory complex in gene transcription through directly binding to Lyn/Akt tyrosine residues.^16^ Lyn can also phosphorylate the Thr308 site of Akt to regulate cell metabolism, proliferation, migration and other physiological functions.^33^ Moreover, IRF5 needs to be transferred to the nucleus through K63 ubiquitination, and then IRF5 can directly bind to the target gene promoters or activate the downstream transcription factors through dimerization after being phosphorylated. The interaction between IRF5 and Lyn/Akt complex can both provides the ubiquitination and phosphorylation in the process of IRF5 nuclear translocation.^12^ Through detecting the cytoplasmic and nuclear IRF5 protein levels, we confirmed that Lyn facilitated nuclear translocation and phosphorylation of IRF5. Studies have shown that IRF5 binds to the cytokine gene promoter to enhance pattern recognition receptor‐induced cytokine expression, and IRF5 also directly binds to the bridging molecule MyD88 and the second messengers IRAK1 and TRAF6 in mouse macrophages to regulate downstream pathways.^34^, ^35^ Furthermore, transcription factors SP1 and PU.1 have been admittedly confirmed to directly bind to glycolytic gene promoters and induce the transcription of key glycolytic enzymes to promote glycolysis.^36^, ^37^, ^38^ The ChIP results also strongly confirmed that IRF5 promoted the binding of SP1 and PU.1 to glycolytic gene promoters, directly suggesting that IRF5 enhanced glycolysis mainly through facilitating glycolytic gene transcription by SP1 and PU.1.

**FIGURE 13:**
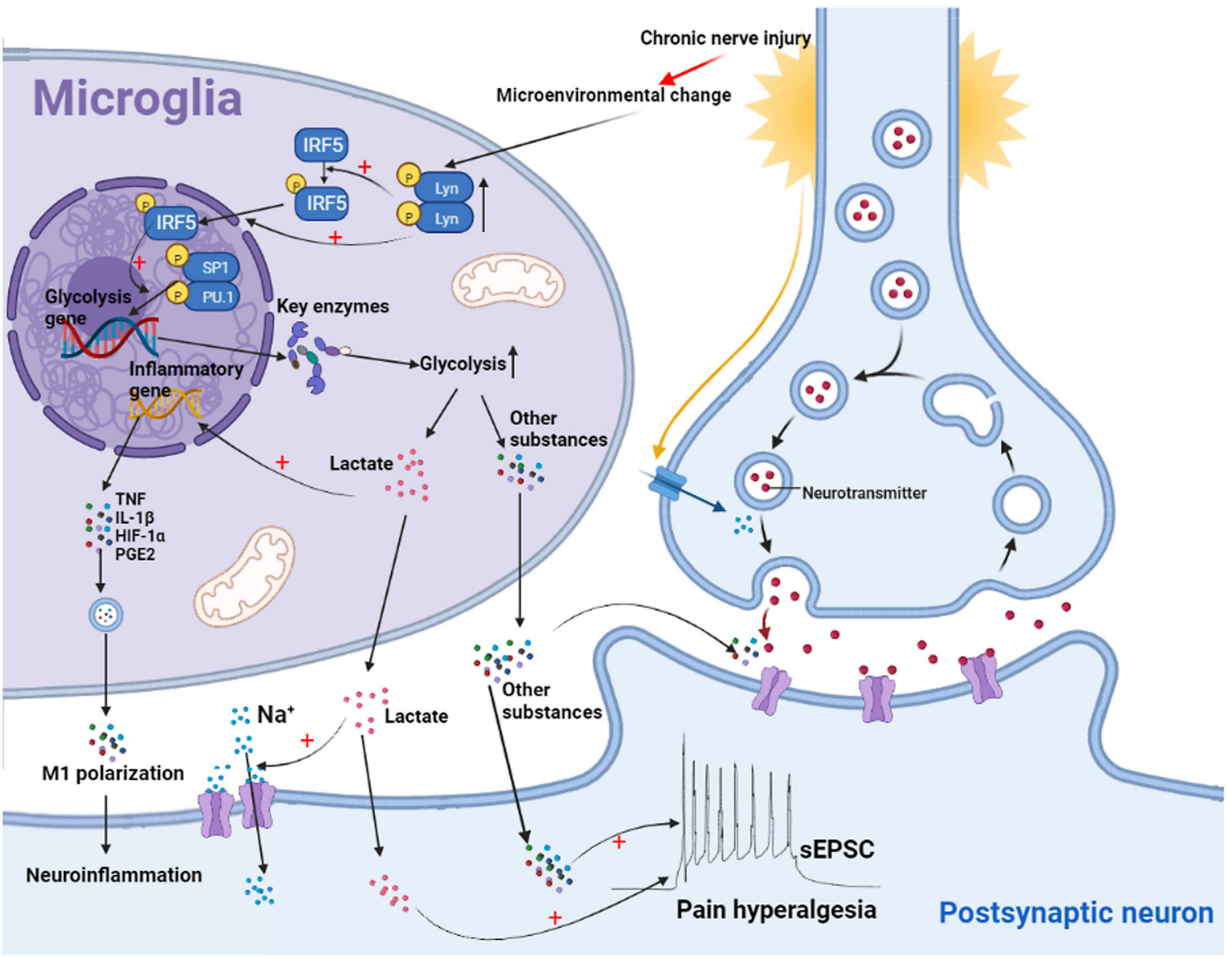
Chronic nerve injury induces the increased expression and phosphorylation of Lyn to mediate IRF5 phosphorylation and nuclear translocation in microglia. Nuclear phosphorylated IRF5 promotes the binding of transcription factors SP1, PU.1 to glycolytic gene promoters and enhances glycolysis of microglia, resulting in the increased production of lactate and other metabolites. Lactate facilitates the proliferation and pro‐inflammatory phenotype transition of microglia to release inflammatory factors such as TNF, IL‐1β, HIF‐1α, PGE2, which contributes vitally to M1 polarization and neruoinflammation. Lactate and other metabolites also facilitates the sEPSC of the postsynaptic neuron. Neruoinflammation and strengthened sEPSC both promote the progression of neuropathic pain. IRF5, interferon regulatory factor 5; TNF, tumour necrosis factor; IL‐1β, interleukin 1β; HIF‐1α, hypoxia inducible factor‐1α; PGE2, prostaglandin E2; sEPSC, spontaneous excitatory postsynaptic currents.

In summary, CCI induces the increased expression and phosphorylation of Lyn to mediate IRF5 phosphorylation and nuclear translocation in microglia. Nuclear phosphorylated IRF5 promotes the binding of transcription factors SP1, PU.1 to glycolytic gene promoters and enhances glycolysis of microglia, resulting in the increased production of lactate and other metabolites. Lactate facilitates the proliferation and pro‐inflammatory phenotype transition of microglia to release inflammatory factors such as TNF, IL‐1β, HIF‐1α, prostaglandin E2 (PGE2), which contributes vitally to M1 polarization and neuroinflammation. Lactate and other metabolites also facilitates the spontaneous excitatory postsynaptic currents (sEPSC) of the postsynaptic neuron. Neruoinflammation and strengthened sEPSC both promote the progression of neuropathic pain (Figure 13). These findings will provide a novel strategy for potential therapies of neuropathic pain.

## AUTHOR CONTRIBUTIONS

**Erliang Kong:** Writing – original draft (equal); writing – review and editing (equal). **Yongchang Li:** Data curation (equal). **Peng Ma:** Formal analysis (equal). **Yixuan Zhang:** Investigation (equal). **Ruifeng Ding:** Methodology (equal). **Tong Hua:** Software (equal). **Mei Yang:** Validation (equal). **Hongbin Yuan:** Conceptualization (lead).

## FUNDING INFORMATION

This work was supported by National Natural Science Foundation of China (82171220 and 81971046), Natural Science Foundation of Henan Province of China (222300420384) and Medical Science and Technology Research Program of Henan Province (SBGJ202003056, SBGJ202102204, LHGJ20220920).

## CONFLICT OF INTEREST STATEMENT

The authors declare that there were no competing interests.

## Data Availability

The data that support the findings of this study are available from the corresponding author upon reasonable request.
